# Maximizing clinical rotation placements for US medical students: exploring an optimization model

**DOI:** 10.1080/10872981.2021.2024488

**Published:** 2022-01-06

**Authors:** Gary L. Beck Dallaghan, Xi Lin, J. Kyle Melvin, Julie Golding, Beat Steiner, Vidyadhar Kulkarni

**Affiliations:** a School of Medicine, University of North Carolina, Chapel Hill, NC, USA; b Operations and Decision Technologies, The University of California-Irvine Paul Merage School of Business, Irvine, CA, USA; c The Department of Family and Community Medicine, UNC Primary Care, Siler City, NC, USA; d Academic Affairs for Application Phase, School of Medicine, University of North Carolina, Chapel Hill, NC, USA; e Statistics and Operations Research, University of North Carolina, Chapel Hill, NC, USA

**Keywords:** Medical student, clinician educator, patient care, operations research, optimizing schedules

## Abstract

**Background:**

For years, US medical schools have relied on community-based, private clinicians to educate medical students. There has been a steady decline in the number of physicians willing to take on medical students in their clinical practices. Recent issues related to the pandemic raise questions about how many patients students should see to have a meaningful clinical experience.

**Methods:**

As part of a 16-week longitudinal clinical experience, medical students spend 2 days each week in a family medicine or internal medicine clinic. As repetition enhances learning, maximizing the number of patients students see is important. Using a mixed integer linear program, we sought to determine the optimal schedule that maximizes the number of patients whom students see during a rotation. Patient visits were collected from January to April 2018 for clinics used by the medical school. By maximizing the minimum number of patients per learner over all non-empty day-clinic combinations, we deliver equitable rotation plans based on our assumptions.

**Results:**

For this pilot study, multiple experiments were performed with different numbers of students assigned to clinics. Each experiment also generated a weekly rotation plan for a given student. Based on this optimization model, the minimum number of patients per student over 16 weeks was 87 (3 patients per day) and actually increased the number of students who could be assigned to one of the clinics from 1 student per rotation to 8 students.

**Conclusions:**

The mixed integer linear program assigned more students to clinics that have more total visits in order to achieve the optimal and fairest learning quality. In addition, by conducting various experiments on different numbers of students, we observed that we were able to allocate more students without affecting the number of patients students see.

## Introduction

A joint report detailing the decline of clinical educators in 2014 highlighted a problem that continues to vex US medical schools [1]. Community-based clinical educators volunteering to teach medical students provide robust experiences for medical students [2,3]. Subsequent to the joint report, a survey of clerkship directors elucidated why clinical educators in the private sector decline requests to teach. The Alliance for Clinical Education (ACE) and Society of Teachers of Family Medicine (STFM) produced recommendations for recruiting clinical educators, focusing on educator preparation and support from the medical school [4,5].

Pediatric clinical educators represented the greatest declining specialty[1]. The pressures to see patients and complete follow-up documentation afterwards consume physicians’ time without the addition of teaching students. Exploratory studies conducted with pediatric community-based educators who currently teach [6] and who have quit teaching [7] identified time and volume of students as reasons to limit or cease teaching.

Although the ACE recommendations address clinical educators’ points [8], other considerations for declining clinical educator involvement were lacking. Other studies identified additional barriers, offering suggestions to remedy their concerns [9,10]. Examples of barriers included limited patient rooms, limited number of workstations, and the clinic culture. These are concerns expressed by clinician educators not only in community-based clinics but also at academic health centers.

Three of the authors have extensive experience scheduling clinical placements. Although we have relied on simply asking clinician educators if they can accommodate an additional student, this is not the ideal approach. When taking into consideration comparability of experiences required by accreditation [11], simply relying on a quick call to schedule a student may result in the student going to a clinic already overbooked with students. Therefore, the numbers of patients available to care for may be insufficient.

Based on evidence that repetition of skills enhances learning and retention [12], we argue a quality clinical learning experience is one that provides a higher number of patient encounters per student per day. This led us to question how to calculate the ideal number of learners a clinic can accommodate without sacrificing the quality of the experience (e.g. patient encounters). Taking into consideration the constraints noted by Melvin et al. [9], one potential method of calculating the appropriate number of students to clinic ratio is employing a mathematical problem-solving technique, known as operations research.

The field of operations research employs analytic techniques to solve complex problems [13]. The scope of problems applying operations research techniques can range from manufacturing plant schedule optimization [14] to electrical brain stimulation [15]. Mixed integer programming is an optimization model that can be formulated through decision variables that have linear relationships. Decision variables are often binary integer variables that can model yes or no decisions as the model is constructed. Using mixed integer programming, various constraints can also be added to the equation, such as attending clinic only on certain days each week. Decision variables and constraints can then be used to address the objective function.

The objective function of this model was to maximize the minimum expected number of patients seen by each student on a given day. We investigate how different plans varied in terms of the student-to-patient ratio based on clinic assignments.

## Materials and methods

### Community-based longitudinal care course

University of North Carolina (UNC) medical students are required to complete a 16-week course designed to provide a longitudinal clinical experience in adult and pediatric medicine during the third year of medical school. At this stage of their education, medical students are expected to have an active role in the clinic encounter, often times independently taking a history and conducting a physical examination followed by reporting findings to the clinical educator. Students may see new and returning patients.

Each student has a different schedule due to preceptor availability. Depending on scheduling, some clinical educators have more than one student they are supervising per clinic period. The general requirements for the course include outpatient training in a family medicine clinic or internal medicine clinic 2 days per week. Each student is assigned to one family medicine or internal medicine clinic for the entire 16 weeks. The student attends clinic the same 2 days each week. One day per week is spent in a pediatrics clinic. The other 2 days are divided between completing a quality improvement project, dedicated didactic sessions, and self-directed learning activities.

### Clinics

Each 16-week period, there are approximately 40 students to schedule in various clinics. The clinics are internal medicine, family medicine, and pediatrics. For the purposes of this exploratory experiment, we included three UNC-affiliated clinics since we had access to clinic records and they regularly teach medical students. Clinics ranged in size from 20 to 52 patient examination rooms. The number of students assigned each period range from 1 to 3 students.

### Mixed-integer program

The formulation phase of the optimization process involved identifying an objective function, constraints on solutions, assumptions, and process descriptions [7]. The aim is to produce a rotation plan for each student to work in the adult medicine clinics. During this period, students are also in a pediatric clinic one day per week; however, data could not be obtained from those clinics for inclusion in the model.

### Constraints

The goal for our model is to provide each student with a five-day schedule that meets the learning requirements. This leads to the identification of the following constraints:

- Number of training day requirements (2 days/week for adult clinics)
- Each student assigned to one clinic for both training days

For every student, the rotation plan for each week was assumed to be identical each week, meaning that student 1 goes to Clinic A Mondays and Wednesdays every week for the 16-week period. We wanted to examine the effect an increase in number of students would have on patient-to-student ratio, which optimizes their experience. Based on current scheduling parameters, we used clinic-defined student capacities as follows:

- Clinic A limited to three students per rotation period
- Clinic B limited to three students per rotation period
- Clinic C limited to one student per rotation period

A linear mixed integer program was constructed to achieve this goal. We specifically defined the learning experience measure as the number of patients divided by the number of students in every clinic on a given day.

### Assumptions

In order to generate a mixed integer program model, some assumptions about the data need to be outlined. As our learning quality measure is patient-student ratio, we needed an estimate of the number of patients who may be seen by students in each clinic on each weekday at baseline. We obtained patient visits from January to April 2018 for our estimates. The raw data contained information of visits, including variables such as patient visit date, patient identification number, and provider number. A frequency table was created to summarize the daily appointment numbers by provider number in each clinic.

We assumed the primary care providers in the clinic by ranking the physician identification number from the most appointments to the least appointments for the 4 months of clinic visits obtained. The data provided included only provider ID number with no way of identifying specific physicians. Therefore, we examined the total number of patient encounters per provider identification number. A noticeable gap was identified for providers with less than 300 patients. We then assumed providers falling below 300 patients were most likely subspecialists providing consultation in the clinic and thus not included in the model.

For each day of the week, we obtained the average number of visits to a physician and used this average to estimate the number of visits for one preceptor in that clinic on each weekday. We further assumed that each student sees an equal proportion of total patients to all preceptors.

The reformulated mixed integer program was solved by AMPL (Mountain View, Ca) using the solver IBM CPLEX (Armonk, NY). Note that UNC Family Medicine, UNC Internal Medicine, and Chapel Hill Internal Medicine were denoted as C1, C2, and C3, respectively. According to the problem setting, parameters were set as follows:

- total days of training *D* was equal to 5;
- required days of training *r* was 2;
- total number of clinics *C* was 3; suggested capacity of students at C1, C2, and C3 was 3, 3, and 1, respectively;
- *p_c_*, the number of preceptors in clinic C is set to be 1 for all clinics.

The average patient estimates per preceptor for all clinic:day combinations are shown in Table 1. The minimum number of patients over all clinic days during the 16-week block (for a given S) is the objective value (N) for the original program. See the supplemental appendix for the mathematical formulation.

**Table 1.** Average patient visit numbers per clinical training site

	Mon	Tue	Wed	Thu	Fri
Clinic 1	77.42	91.79	60.11	76.53	59.53
Clinic 2	69.44	75.89	89.33	42.11	68.89
Clinic 3	182.00	178.60	161.80	168.00	166.40

### Model confirmation

To explore if the linear mixed integer program was accurate, we compared simulation results to actual schedules. We looked only at numbers of students assigned to clinics as that was the primary purpose for this model.

This study was reviewed by the UNC Institutional Review Board. The study was approved (IRB No. 18–2771).

## Results

Computer simulations were performed experimenting with different numbers of students to be assigned to the three clinics. The initial simulation included seven students as that reflected the actual number of students assigned to these three clinics. Subsequent experiments were conducted by adding one student until the model failed to find a solution, which was after 15 students were included in the model. For each simulation, the number of students and the estimated number of patients seen by one student in every clinic on a given day were calculated. Weekly rotation plans for each student were also generated by the model. Finally, we calculated the projected number of patients seen by each student over the entire training period of 16 weeks.

Table 2 summarizes the number of students assigned to every clinic on each day of the week. We noticed that when *S* is small, student-to-patient ratios are distributed more evenly; for larger *S* placements per clinic, the program assigned more students to C3. It is also noteworthy that the number of students in C1 or C2 on a given day stays unchanged as additional students were assigned, whereas C3 becomes more crowded as student placements increase.

**Table 2.** Students and patient-to-student ratio for each clinic

	**Clinic 1**					**Clinic 2**					**Clinic 3**				
**S = 7**	Mon	Tue	Wed	Thu	Fri	Mon	Tue	Wed	Thu	Fri	Mon	Tue	Wed	Thu	Fri
1	77.42		60.11												
2		45.90		76.53											
3		45.90			59.53										
4						69.44		44.66							
5							75.89		42.11						
6								44.66		68.89					
7											182.00				166.40
**S = 8**															
1	77.42		60.11												
2		45.90		76.53											
3		45.90			59.53										
4						69.44		44.66							
5							75.89		42.11						
6								44.66		68.89					
7											182.00	89.30			
8												89.30		168.00	
**S = 10**															
1	77.42		60.11												
2		45.90		76.53											
3		45.90			59.53										
4						69.44		44.66							
5							75.89		42.11						
6								44.66		68.89					
7											60.67	89.30			
8											60.67		161.80		
9											60.67				83.20
10												89.30			83.20
**S = 15**															
1	77.42		60.11												
2		45.90		76.53											
3		45.90			59.53										
4						69.44		44.66							
5							75.89		42.11						
6								44.66		68.89					
7											45.50		53.93		
8											45.50		53.93		
9											45.50			42.00	
10											45.50			42.00	
11												44.65		42.00	
12												44.65		42.00	
13												44.65			55.47
14												44.65			55.47
15													53.93		55.47

S = student

Patient-to-student ratios are indicated for each student (S) by day of the week and by clinic for different numbers of students. All the values after assigning 15 students are above the optimized value of 42.

The patient-to-student ratios differ from each other for smaller numbers of students assigned to a clinic. For instance when S = 7, the largest patient-to-student ratio is equal to 182 but the smallest is equal to 42.11. However, for 15 students, the largest ratio equals to 77.42 and the smallest one equals to 42, indicating greater comparability for students across all three clinics (Table 2).

For 7, 8, or 10 students, the objective value equals 42.11. That is, the objective value suggests that every patient-to-student ratio is expected to be greater than 42.11 per day over the course of 16 weeks. Thus, with two training days the objective value is increased to 84.22. In the case of 15 students, the objective value drops by 0.11 to 42. Hence, we can assign 15 students in these three clinics without compromising the minimum number of patients over all clinic days during the 16-week block for a given student. Note this is a proportion of patients-to-students per day and not intended to imply that a student will see 42 patients per day.

Based on these findings, we were able to create schedule template for students and clinics (Table 3). Over the course of 16 weeks, the minimum number of patients obtained from optimizing the patient-to-student ratio was calculated at 87.50 patients per student, which is roughly 3 patients per clinic day. Likely the students would see more patients than this, but the model indicates that this is the minimum number required to optimize the schedule.

**Table 3.** Rotation plans and total number of patients seen by every student

	Mon	Tue	Wed	Thu	Fri	Patients Seen
S1	C3	–	–	–	C3	100.97
S2	–	–	–	C3	C3	97.47
S3	–	–	C2	C2	–	86.77
S4	C2	C2	–	–	–	145.33
S5	C1	C1	–	–	–	123.31
S6	–	–	C1	C1	–	136.64
S7	C3	C3	–	–	–	90.15
S8	–	C3	–	–	C3	100.12
S9	–	–	C2	–	C2	113.55
S10	–	C3	C3	–	–	98.58
S11	–	C1	–	–	C1	105.42
S12	C3	–	–	C3	–	87.50
S13	–	–	C3	C3	–	95.93
S14	–	C3	C3	–	–	98.58
S15	C3	–	–	C3	–	87.50

S = student; C = clinic

Prior to the completion of our optimization model, Clinic 3 allowed only one student to rotate per course. However, when the results of this model were presented to the course coordinator, Clinic 3 had agreed to take the exact number of students our model identified, providing initial verification of the results.

## Discussion

Applying a mixed integer program model optimized the number of students per patient ratio for the three clinics. The model indicated that Clinic 3 could actually accommodate a greater number of students without compromising the patient-to-student ratio for clinical experiences.

Additional benefits emerge from using this mixed integer program model. The first is that we identified a smaller variance in patient-to-student ratio across clinical placements. With requirements from accreditation to catalog comparable experiences across clinical training sites [11], this is one way to meet this expectation. Our pilot included three clinics, but additional clinics could be added to calculate these numbers. The other benefit is the ability of the model to generate a schedule for students that can optimize their patient care experiences.

From the estimated rotation plans, our mixed integer program scheduled students for 2 days of training per week, and they were assigned to the same clinic. In addition, we were able to calculate how many students were assigned to each clinic for each experiment. When we increased the number of students assigned to Clinic 1 or Clinic 2, the patient numbers remained stable, which was equal to the suggested capacity of three students. Meanwhile, all extra students were assigned to Clinic 3. This was reasonable since the mixed integer program strives to enable students to see more patients.

Simulations on different combinations of preceptors per clinic were performed as well, and we focused on how the student-clinic assignments were affected. If all three clinics had the same number of preceptors, the final calculation did not change the optimal rotation plans as outlined above. These results confirm clinic scheduling options suggested by Melvin et al [9].

Clinicians have expressed concerns about the impact of student education with regard to patient numbers [6,16,17]. Our model sought to maximize patient-to-student ratios based on the minimum average patient per preceptor in a clinic. In so doing, the impact on preceptor productivity should be minimized. Sairenji and colleagues found that teaching students compared to not teaching had negligible impact on calculated relative value units[18]. Bhagwat and colleagues also found that multiple students placed at a clinic did not extend the length of the work day in a significant manner[19].

The success of this model still depends on the willingness of physicians to teach. Our model focused on the patient-to-student ratio as an indicator of a quality patient care experience, not specifically the quality of the clinician educator. Based on research related to building and enhancing clinical skills [12], more practice has been shown to improve skills. We were fortunate to find that in all three clinics, the optimal number of students assigned to the clinics matched our model. Student evaluations have yet to be reviewed to determine if teaching by the preceptors was impacted by the influx of learners.

### Limitations

One of the limitations of this study is that a mix of patient conditions was not included in the calculations. Students should be exposed to patients of varying complexity, but these data were not included. However, the optimization model can be modified to incorporate additional constraints to provide an optimal mix of patients. Since our initial goal was to determine the optimal number of students to schedule at a clinic, this was not explored for this initial pilot. Additionally, this study was limited to three clinics. Further studies are merited that would include the broad array of ambulatory clinics students are assigned to so that the model can be further refined. For a pilot, the results were promising and provide a framework for further adoption.

## Conclusion

The mixed integer program provided medical students with weekly rotation plans that provide guidance for an equitable patient care experience. In general, this program assigned more students to clinics that have more total visits in order to achieve the optimal and most fair overall clinical learning experience. This model can provide guidance to clerkship directors to determine an equitable patient care experience across multiple clinical training sites. In addition, by conducting various experiments on different numbers of students, we observed that we were able to allocate more students without affecting their overall ratio of students to patients.

## Supplementary Material

Supplemental Material

## Data Availability

Data are available upon request.
