# Supplementary material for: Maximizing clinical rotation placements for US medical students: exploring an optimization model
Source: Med Educ Online. 2022 Jan 6;27(1):2024488. doi: 10.1080/10872981.2021.2024488 (PMC8741226; doi:10.1080/10872981.2021.2024488)
Supplement: Supplemental Material [file ZMEO_A_2024488_SM6562.docx]

Supplemental Appendix: Mathematical Formulation

**Mixed Integer Program Introduction**

Linear programming is an optimization technique that has a variety of applications. The method of problem solving for linear programming may be formulated using decision variables that have a linear relationship. Therefore, needed variables for a linear programming model include a set of decision variables, a set of linear constraints on those variables, and a linear objective function to maximize or minimize. An example might be how to maximize vaccine production in one month from three different factories whose production outputs and costs vary.

Decision variables are typically in the form of a Yes or No (1 or 0) response. These variables also need to have lower and upper bounds applied to them, in the case of our example it would be 30 days (0 to 30). Constraints have to be linear in nature, and for the vaccine production example it would be the number of vaccines produced by a particular factory that are equal to or exceed the goal amount of total vaccines to be produced. Bounds can also be placed on the constraints, such that no factory would produce twice as much as one of the others.

A problem becomes a mixed integer program when variables are not continuous and the decision variables place more constraints. In the vaccine production example, if it was determined that only two factories could be working at the same time, the question then becomes what the optimal combination of factories working to maximize production of the vaccine. Due to this problem now having a non-linear objective function, the problem must be reformulated to incorporate a minimization process that results in a linear function.

Our model aims to accommodate *S* students into *C* clinics for a learning period of *D* days. Students are required to receive training for *r* of the total *D* days. The objective is to maximize the smallest number of patients seen by each student over all clinic:day combinations.

**Notations**

- - - Set of students: S = {1*,* 2*, ..., S*}.
    - Set of clinics: C = {1*,* 2*, ..., C*}.
    - Set of days: D = {1*,* 2*, ..., D*}.

**Parameters**

- - - *r* is the required days of training for each student in family medicine clinics. Integer.
    - *a_cd_* is the number of estimated patients for one preceptor in *clinic c* ∈ C on *day d* ∈ D. Integer.
    - *l_c_* is the suggested capacity of *clinic c* ∈ C. Integer.
    - *p_c_* is the number of preceptors in clinic *c*.

**Decision Variables**

- - - *X* is a three-dimensional matrix:

*X_scd_* = 1 if *student s* ∈ S is assigned to *clinic c* ∈ C on *day d* ∈ D, 0 otherwise.

- - - *y_sc_* = 1 if *student s* ∈ S is assigned to *clinic c* ∈ C, 0 otherwise.

**Original Program**

Σ*s*∈*S X_scd_* is the number of students in *clinic c* on *day d*. *p_c_* · *a_cd_* is the total number of patients to *clinic c* on *day d* that will be seen by students. Therefore, *p_c_* * *a_cd_/*Σ*s*∈*SX_scd_* is the number of patients seen by each student working in *clinic c* on *day d*.

For every feasible plan, we consider the minimum over all *(c, d)*. We find the feasible plan that has the largest minimum.

max min *p_c_* · *a_cd_*

X (c,d):Σ*s*∈*S X_scd_>*0 *s*∈*S X_scd_*

**Constraints**

Number of training day requirements:

∀*s* ∈ S Σ Σ *X_scd_* = *r* (1)

*c*∈C *d*∈D

On a given day, a student should be in no more than one clinic:

∀*s* ∈ S*, d* ∈ D : Σ *X_scd_* ≤ 1 (2)

*c*∈C

One student can only be assigned to one and only one clinic:

∀*s* ∈ S : Σ *y_sc_* = 1 (3)

*c*∈C

If the student is assigned to *clinic 3*, the student will remain at that clinic for two days every week; otherwise, the student will spend no time in that clinic:

∀*s* ∈ S*,* ∀*c* ∈ C : Σ *X_scd_* ≤ *r* · *y_sc_.* (4)

*d*∈D

Equation (5) considers the plans that make full use of the given capacity:

∀*c* ∈ C : Σ *y_sc_* ≥ *l_c_* (5)

*s*∈S

**Reformulation into Mixed Integer Linear Program**

Due the non-linear objective function, the original program needed to be reformulated as follows:

**New Objective**

min *z*

**Constraints**

(1) – (5)

Ɐ*c*∈*C*, *d*∈*D*:Σ*_s_*_∈_*_S_X_scd_*<*z***p_c_***a_cd_*
